# Impact of diabetes mellitus and co-morbidities on mortality in patients with COVID-19: A single-center retrospective study

**DOI:** 10.15537/smj.2023.44.1.20220462

**Published:** 2023-01

**Authors:** Anees A. Sindi, Wail A. Tashkandi, Mohammed W. Jastaniah, Mohammed A. Bashanfar, Ahmed F. Fakhri, Fahad S. Alsallum, Hamdan B. Alguydi, Alyaa Elhazmi, Talal A. Al-Khatib, Maha M. Alawi, Ibrahim Abushoshah

**Affiliations:** *From the Department of Anesthesia and Critical Care (Sindi, Abushoshah), from the Department of Surgery (Tashkandi), from the Department of Otolaryngology-Head Neck Surgery (Al-Khatib), from the Unit of Infection Control & Environmental Health (Alawi), Department of Medical Microbiology & Parasitology (Alawi), Faculty of Medicine (Jastaniah, Bashanfar, Fakhri, Alguydi, Alsallum), King Abdulaziz University and King Abdulaziz University Hospital, Jeddah, Saudi Arabia, from the College of Medicine (Elhazmi), Alfaisal University, and from the Research Center (Elhazmi), Dr. Sulaiman Al-Habib Medical Group, Riyadh, Kingdom of Saudi Arabia.*

**Keywords:** diabetes mellitus, COVID-19, SARS-CoV-2, HbA1c

## Abstract

**Objectives:**

To describe the effect of diabetes mellitus (DM) on clinical outcomes of patients admitted with COVID-19 infection.

**Methods:**

We carried out a single center, observational, retrospective study. We included adult patients with laboratory-confirmed diagnosis of COVID-19 admitted to a tertiary hospital in Jeddah, Saudi Arabia, from April 2020 to December 2020. Electronic medical records were reviewed for demographics, clinical status, hospital course, and outcome; and they were compared between the patients with or without DM.

**Results:**

Out of 198 patients included in the study, 86 (43.4%) were diabetic and 112 (56.5%) were non-diabetic. Majority of the patients were males 139 (70.2%) with a mean age of 54.14±14.89 years. In-hospital mortality rate was higher in diabetic patients than in non-diabetic patients (40 vs. 32; *p*=0.011). The most common comorbidity was hypertension (n=95, 48%) followed by ischemic heart disease (n=35, 17.7%), chronic kidney disease (n=17, 9.6%), and bronchial asthma (n=10, 5.1%).

**Conclusion:**

The risk of SARS-CoV-2 infection is higher among diabetic patients; particularly, those with preexisting co-morbidities or geriatric patients. Diabetic patients are prone to a severe clinical course of COVID-19 and a significantly higher mortality rate.

**C**oronavirus disease-19 (COVID-19) pandemic caused by novel coronavirus, severe acute respiratory syndrome-coronavirus-2 (SARS-CoV-2), affected almost all the countries including Saudi Arabia.^1^ Diabetes mellitus (DM) is a well-documented predictor of mortality in previous respiratory viral outbreaks, such as influenza A (H1N1), and SARS-CoV.^2-4^ A growing body of evidence suggests that patients of COVID-19 with DM are more often accompanied by severe or critical disease varying from 14-32%.^5-7^ Wang et al^8^ found that significantly higher number of COVID-19 patients with comorbidities were admitted to the intensive care unit (ICU) than those without comorbidities (72% vs. 37%).

Innate immune system, the first line of defense against SARS-CoV-2, is compromised in patients with DM which is known to impair the process of chemotaxis and phagocytosis in polymorphonuclear neutrophils (PMNs).^9^ Diabetes mellitus has been shown to be a pro-inflammatory condition due to the excess production of the cytokines. Coronavirus disease-19 patients, with pre-existing DM, have shown to have higher serum interleukin-6 (IL-6), C-reactive protein (CRP), and ferritin levels than those without DM.^10^ Cariou et al^11^ found out that 1 in 5 patients with DM were intubated and mechanically ventilated within the same length of time.

Good glycemic control reduces mortality and morbidity, as illustrated by Zhu et al^12^ in their cohort of 7,333 COVID-19 patients. According to the Centers for Disease Control and Prevention, patients with DM are at up to 10 times higher risk of death with COVID-19 infection.^13^ Although many studies have been carried out in this aspect, limited studies are available from Saudi Arabia. Therefore, we planned this study to assess the clinical characteristics and outcomes of COVID-19 patients with co-existing DM.

## Methods

We carried out a single center, observational, and retrospective study among patients with laboratory-confirmed diagnosis of COVID-19 at a tertiary care center located in Jeddah, Saudi Arabia.

We included all adult patients admitted in the hospital with COVID-19 infection. Only patients with the confirmed diagnosis of COVID-19 were included in this study when nasal and pharyngeal swabs showed a positive result on real-time reverse-transcriptase polymerase chain reaction (RT-PCR) for SARS-CoV-2. Data were collected from COVID-19 database for the patients hospitalized from April 2020 to December 2020, regardless of requirement for ICU care. Patients with missing or incomplete information were excluded from the study. An approval was obtained from the institutional ethics committee before starting data collection.

We analyzed electronic medical records to obtain information on recent exposures, signs, and symptoms. Baseline parameters like age, gender, body mass index (BMI), history of smoking, presence of comorbidities, blood pressure (BP), heart rate (HR), respiratory rate (RR), and oxygen saturation (SpO_2_) levels were collected for all the patients. Details of the following laboratory results were collected: complete blood count (CBC), HbA1c, liver and renal function tests (LFT & RFT), C-reactive protein (CRP), procalcitonin (PCT), lactate dehydrogenase (LDH), creatine kinase (CK), D-dimer, ferritin, and troponin. All the investigations were carried out at the time of admission. We also evaluated the clinical outcomes (discharged alive and in-hospital mortality).

### Statistical analysis

Statistical analyses were carried out using the Statistical Package for the Siocial Sciences, version 28.0 (IBM Corp., Armonk, NY, USA). The data was managed for outliers and duplication. Comparison of mean was carried out using 2 independent t-tests. Association was assessed using Chi-sqaure/Fisher’s exact tests. In-hospital mortality was analyzed using Kaplan-Meier estimate and comparison of the mortality curves was reported using log rank (Mantel Cox). Binary logistic regression was carried out to arrive at a prediction model using step wise method in choosing the best predictor. Statistical significance was set at a *p*-value of <0.05.

## Results

A total of 198 patients fulfilled the inclusion criteria and were included in the study; out of them, 86 (43.4%) were diabetic and 112 (56.6%) were non-diabetic. Majority of the patients were males 139 (70.2%) with a mean age of 54.14±14.89 years. The mean BMI was 31.38±30.14 with the majority being overweight (n=75, 37.9%). The most common comorbidity was hypertension (HTN) in 95 (48%) cases followed by ischemic heart disease (IHD) in 35 (17.7%), chronic kidney disease (CKD) in 17 (9.6%), and bronchial asthma in 10 (5.1%) patients. Demographic details and other patient characteristics are presented in the Table 1.

**Table 1:** - Characteristics of all the study subjects (N=198).

Variables	n (%)
***Gender***	
Male	139 (70.2)
Female	59 (29.8)
***Health care worker***	
Yes	19 (9.4)
No	179 (90.6)
***Age, mean±SD***	54±14.89
17-35 years	22 (11.1)
36-50 years	51 (25.8)
>50 years	125 (63.1)
***DM***	
Yes	86 (43.4)
No	112 (56.6)
Active smokers	9 (4.5)
***Body mass index, mean±SD***	31.38±30.14
Normal	74 (37.8)
Underweight	1 (0.5)
Overweight	75 (37.9)
Obese	48 (24.2)
***Co-morbidities present prior to admission***	
Hypertension	95 (48.0)
Ischemic heart disease	35 (17.7)
Chronic kidney disease	19 (9.6)
Asthma	10 (5.1)
Chronic obstructive pulmonary disease	3 (1.5)
Chronic liver disease	3 (1.5)
Chronic Lung disease	2 (1.0)
HbA1c (mmol/mol), mean±SD	8.65±3.19
***Oxygen therapy (on hospital arrival)***	
Required	96 (48.5)
Not required	102 (51.5)

Values are presented as numbers and precentages (%). SD: standard deviation, DM: diabetes mellitus, HbA1c: hemoglobin A1C

Table 2 shows the comparison of demographic and clinical profiles between the diabetic and non-diabetic patients. Diabetic patients were older than non-diabetic patients (60.89±12.00 vs 49.43±15.03 years; *p*<0.001). Significantly greater number of diabetic patients had co-morbidities (HTN, IHD, and CKD) than non-diabetic patients. At the time of admission, diabetic patients had higher RR (25.31±7.39 vs. 21.97±4.7; *p*<0.001) and lower SpO_2_ (90.23±13.16 vs. 93.8±10.20; *p*=0.041) than non-diabetic patients. They also had longer duration of hospital stay (median [IQR]: 13 [10] vs. 11 [9]; *p*=0.027) than non-diabetic patients.

**Table 2:** - Demographic and clinical profile of diabetic and non-diabetic patients at the time of admission.

Variables	Diabetic (n=86)	Non-diabetic (n=112)	*P*-values
Age in years, mean±SD	60.89±12.00	49.43±15.03	<0.001
Male	60 (69.7)	79 (70.5)	0.907
Active smoker	3 (3.5)	6 (5.3)	0.531
Fever	66 (76.8)	86 (76.8)	0.995
Cough	58 (67.4)	74 (66.1)	0.839
Dyspnea	62 (72.1)	64 (58.9)	0.030
Headache	6 (7.2)	13 (11.6)	0.273
Altered consciousness	8 (9.3)	6 (5.3)	0.283
Hypertension	69 (83.1)	26 (23.2)	<0.001
Ischemic heart disease	28 (32.6)	7 (6.3)	<0.001
Chronic kidney disease	17 (19.8)	2 (1.8)	<0.001
Length of hospital stay	13 (10.0)	11 (9.0)	0.027
Mean arterial pressure, mean±SD	80.51±20.01	92.71±15.76	0.001
Systolic blood pressure, mean±SD	139.58±23.98	132.31±21.48	0.059
Diastolic blood pressure, mean±SD	74.68±12.88	79.25±13.11	0.040
Heart rate, mean±SD	92.64±21.06	90.85±16.62	0.535
Respiratory rate, mean±SD	25.31±7.39	21.967±4.7	<0.001
Oxygen saturation, mean±SD	90.23±13.16	93.8±10.20	0.041
PCO2, mean±SD	36.42±13.75	40.27±18.0	0.325
HbA1C, mean±SD	9.64±3.32	6.56±1.89	<0.001
Hemoglobin, mean±SD	12.21±237	12.96±2.65	0.056
Ph value, mean±SD	7.31±0.13	7.37±0.10	0.047

Values are presented as numbers and precentages (%). SD: standard deviation, PCO2: partial pressure of carbon dioxide, HbA1C: hemoglobin A1c, pH: potential of hydrogen

Table 3 highlights various laboratory parameters among both the groups. Laboratory parameters of diabetic patients revealed significantly higher median WBC (*p*=0.006), neutrophil (*p*<0.001), CRP (*p*=0.004), PCT (*p*<0.001), creatinine (*p*<0.001), D-dimer (*p*=0.008), and troponin levels (*p*<0.001).

**Table 3:** - Laboratory parameters on hospital arrival among diabetic and non-diabetic group.

Laboratory parameters	Diabetic	Non-diabetic	*P*-values
WBC (X10^9^/L)	8.05 (5.7)	6.26 (3.87)	0.006
Neutrophils (X10^9^/L)	6.49 (5.18)	4.09 (3.85)	<0.001
Lymphocytes (X10^9^/L)	1.46 (.74)	1.17 (0.93)	0.154
Platelet count (X10^9^/L)	252 (176.25)	216.5 (128)	0.045
CRP (mg/L)	100.85 (0)	56.3 (31.05)	0.004
AST (U/L)	51 (0)	37.5 (39.5)	0.479
(g/L)	170 (0)	77.88 (42.5)	<0.001
LDH (U/L)	561 (0)	295 (340.8)	0.691
Bilirubin	15.5 (0)	7.5 (6.0)	0.425
Lactate	1.3 (0)	1.15 (0.61)	0.429
Procalcitonin	0.49 (0)	0.06 (0.25)	<0.001
Creatinine-kinase	965 (0)	76.5 (58.5)	0.212
D-dimer	1.39 (0)	1.79 (2.98)	0.008
Ferritin	5320.16 (0)	237.7 (1326.3)	0.052
Troponin	0.34 (0)	0.015 (0.016)	0.009

Values are presented as medians and interquartile ranges (IQR). WBC: white blood count, CRP: C-reactive protein, AST: aspartate aminotransferase, LDH: lactate dehydrogenase

Table 4 represents the risk factor analysis for diabetic patients. First, univariate analysis was carried out using appropriate test to determine which variable were significant represent the risk factor for diabetic patients. Then significantly associated variables were analyzed using multivariate analysis. The variables that were considered included: age, HTN, shortness of breath, IHD, CKD, WBC, CRP, and PCT. Among these variables, only age was significant in the adjusted analysis.

**Table 4:** - Risk factors analysis.

Risk factors	Unadjusted analysis		Adjusted analysis	
	OR (95% CI)	*P*-values	OR (95% CI)	*P*-values
Age	0.941 (0.919-0.964)	<0.001	0.936 (0.887-0.988)	0.017
Hypertension	0.074 (0.037-0.148)	<0.001	0.255 (0.055-1.171)	0.079
Shortness of breath	0.516 (0.283-0.942)	0.031	0.964 (0.172-5.410)	0.967
Ischemic heart disease	0.138 (0.057-0.336)	<0.001	0.237 (0.023-2.427)	0.225
Chronic kidney disease	0.074 (0.017-0.329)	<0.001	0.000	0.999
C-reactive protein	0.993 (0.988-0.998)	0.006	0.998 (0.985-1.010)	0.694
Procalcitonin	0.369 (0.205-0.665)	0.011	0.539 (0.204-1.424)	0.213

OR: odds ratio, CI: confidence interval

**Figure 1:**
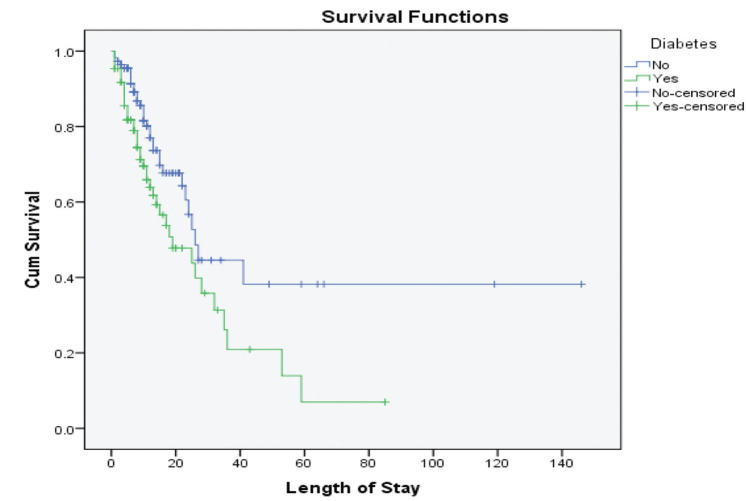
- Survival time for diabetic and non-diabetic patients.

Out of a total 86 diabetic patients, 46 (53.5%) were discharged alive while 80 (71.4%) out of 112 non-diabetic patients were discharged successfully. In-hospital mortality rate was higher for diabetic patients 40 (46.5%) as compared to non-diabetic patients 32 (26.4%). There was a significant relationship between the diabetics and outcome variables (X^2^ (N=198)=6.53, *p*=0.011). In Figure 1, Kaplan-survival curves show that diabetes patients had an earlier in-hospital mortality when compared to non-diabetic patients. Median survival time for diabetic group was 19 (7.8-30.11) days whereas for the non-diabetic group it was 26 (21.9-30.03) days (*p*=0.011).

With the increasing age, the risk of mortality was increasing significantly and in the age group ≥50 years, 43.2% patient died (*p*=0.006). Other co-morbidities including HTN, and IHD were significantly associated with the mortality in COVID-19 disease.

Death percentage was 28.1% (9 deaths out of 32) in patients without any co-morbidity which increased to 28.1% (16/57) with one co-morbidity, 34.9% (15/43) with 2 co-morbidities, 48.3% (14/29) with 3, 47.1% (8/17) with 4, 41.7% (5/12) with 5, 66.7% (2/3) with 6, and 60% (3/5) with 7 co-morbidities. The percentage of death increased gradually with the increasing number of comorbidities; however, it was not statistically significant (*p*=0.347; Table 5).

**Table 5:** - Mortality analysis with age group and comorbidities.

Parameters	Total	Outcomes		Death percentages	*P*-values
		Alive	Death		
***Age group (years)***					
17-35	22	20	2	9.1%	0.006
36-50	51	35	16	31.4%	
≥51	125	71	54	43.2%	
Diabetes	86	46	40	46.5%	0.009
Hypertension	95	50	45	47.4%	0.002
Ischemic heart disease	35	17	18	51.4%	0.041
COPD	3	2	1	33.3%	0.912
CKD	19	9	10	52.6%	0.121

COPD: chronic obstructive pulmonary disease, CKD: chronic kidney disease

## Discussion

In the present study of 198 patients, 86 patients had DM with a mean age of 54±14.89 years and male predominance (70.2%). Previous studies also showed a similar involvement of elder patients (50-70 years), and a male preponderance.^14,15^ In spite of some studies showing gender differences in COVID-19 cases and in their fatality rate, a growing number of studies showed no gender differences in SARS-CoV-2 infections.^16,17^ In our study, 37.9% were overweight. According to a meta-analysis both higher BMI and obesity were associated with the poorer patient outcomes (ICU admission, severe COVID-19, use of mechanical ventilation, hospital admission, and mortality) in COVID-19 adult patients.^18^ Excessive ectopic fat deposition increases susceptibility to severe infection and subsequent multi-organ failure by potentiating the immune dysregulation.^19^

Hemoglobin A1c is a stable index of long-term glucose control and provides an average value of the past 3 months.^20^ In our study, the mean HbA1c in the diabetic group was 9.64±3.32. A previous population-based study showed a higher COVID-19-related mortality in people with HbA1c >7.5%, and another identified that mortality in people with type 1 and type 2 DM was independently related to the HbA1c.^21,22^ The proposed mechanisms include hyperglycemia induced inhibition of intracellular destruction of microbes, neutrophil chemotaxis, and phagocytosis; thereby, providing higher affinity for cellular binding and effective virus entry, and reducing viral clearance.^23^ Furthermore, it is believed that SARS-CoV-2 uses ACE2 as entry receptors, which are present on the islets of Langerhans. This can cause mild to fulminant damage to these cells, leading to clinical states varying from mild hyperglycemia to life-threatening diabetic ketoacidosis.^24^

On risk factor analysis, age, hypertension, shortness of breath, IHD, CKD, high WBC, CRP, and PCT levels at admission were found to be more common in the diabetic patients than in non-diabetic patients; however, on adjusted analysis, only the age was found to be a significant risk factor. In the present study, 48.5% patients required oxygen therapy. This finding is in line with a previous study in which 47.9% of patients required mechanical ventilation.^25^ In the present study, 3.5% of diabetic patients were smokers. Smoking is associated with a poor prognosis in COVID-19 patients due to harmful impact of tobacco on the lungs and the immune system with consequent poor response to microbial infections.^26^ A previous systematic review showed the possible adverse impact of smoking on disease severity and outcome of hospitalized COVID-9 patients.^27^

In the present study, all the laboratory parameters were higher in diabetic than non-diabetic patients. These results indicated that biochemical parameters may be considered as critical factors the severity and the progression in COVID-19 infected diabetic patients. Ferritin is increased in many viral or bacterial infections.^28^ Previous studies have also reported that elevated levels of ferritin might be associated with a composite poor outcome.^5^ In our study, serum ferritin levels were significantly higher in diabetic than in non-diabetic patients (5320.0 vs. 237.3). Similarly, D-dimer levels were also significantly higher in diabetic patients than in non-diabetic COVID-19 patients (*p*=0.008). D-dimer is a biomarker of active coagulation and thrombin formation and an increase in D-dimer levels in COVID-19 is helpful to identify pulmonary complications, and risk of thromboembolism.^29^ C-reactive protein, an acute phase reactant, increases in presence of inflammation and an elevated CRP level is associated with the severe COVID-19 infections.^5,30^ We also found significantly higher CRP levels in COVID-19 positive diabetic patients (*p*=0.004) in our study. Previous meta-analysis study has also shown the remarkably higher serum CRP and D-dimer levels in COVID-19 diabetic patients.

Clinical outcomes of COVID-19 infected patients depend upon many factors like therapies received during hospitalization as well as associated comorbidities. Diabetes mellitus has been identified as an independent risk factor for developing respiratory infections.^31^ Patients with underlying comorbidities had a longer duration of ICU, hospital stay, and have worse outcomes.^32^ In our study also, in-hospital mortality rate was higher for diabetic patients (46.5%) as compared to 28.6% in non-diabetic patients. Also, diabetes patients had an earlier in-hospital mortality than the non-diabetic patients (median survival: 19 [7.8-30.11] vs. 26 [21.9-30.03] days; *p*=0.011). In a recent meta-analysis, it was found that diabetes was associated with increased severity and mortality. They found DM as the best predictor of a worse COVID-19-related outcome.^33^ The results of another meta-analysis showed that patients with COVID-19 who have DM have a 2-fold higher mortality risk.^34^

Approximately 30-50% of COVID-19 patients reported having at least one comorbidity, including hypertension, diabetes, COPD, cardiovascular diseases, hepatic, and renal diseases.^35^ In our study, 48% had hypertension at the time of admission while 43.5% had DM, 17.7% had IHD, and 9.6% had CKD. These finding are consistent with the results of a previous study showing hypertension (32%) and IHD (9.2%) as the most common comorbidities.^25^ Previous studies and meta-analyses have shown that hypertension, DM, IHD, CKD, COPD, and active malignancies all were associated with higher mortality in COVID-19 patients.^7-10,33,34^ Similarly, in our study, age, hypertension, DM, and IHD were significantly associated with higher mortality; however, only age was an independent risk factor on multivariate analysis as mortality increased with increasing age (*p*=0.006). This difference can be explained by the small sample size to prove such association.

In our study, we found that risk of mortality increased with increasing number of associated co-morbidities in COVID-19 patients. Similar results were seen in other studies also.^36,37^ In a retrospective analysis of 121,342 adult patients, Yoshida et al^37^ found the increased risk of mortality with higher Charlson comorbidity index (CCI) and CCI components and this risk was more in women than in men.

### Study limitations

First, it was a unicentric study with potentially limited external validity. Secondly, it was a retrospective study with some inherent drawbacks such as missing information. Also, the sample size was relatively small which might have affected the final results; especially, when we could not rule out confounding effect due to small number of cases in each sub-group. In light of that, we recommend further validation of these findings with a large sample size in a future study.

In conclusion, our study highlights the complex relationship between the COVID-19 and DM. The risk of SARS-CoV-2 infection is higher for diabetic patients, particularly those with preexisting comorbidities and elder patients. We found DM as an independent risk factor for higher mortality among patients hospitalized for COVID-19 disease.
